# Transcriptional profile of *Paracoccidioides* induced by oenothein B, a potential antifungal agent from the Brazilian Cerrado plant *Eugenia uniflora*

**DOI:** 10.1186/1471-2180-13-227

**Published:** 2013-10-12

**Authors:** Patrícia Fernanda Zambuzzi-Carvalho, Patrícia Kott Tomazett, Suzana Costa Santos, Pedro Henrique Ferri, Clayton Luiz Borges, Wellington Santos Martins, Célia Maria de Almeida Soares, Maristela Pereira

**Affiliations:** 1 Departamento de Bioquímica e Biologia Molecular, Laboratório de Biologia Molecular, Instituto de Ciências Biológicas, ICBII, Campus II, Universidade Federal de Goiás, C.P. 131, 74001-970 Goiânia, GO, Brazil; 2 Laboratório de Bioatividade Molecular, Instituto de Química, Universidade Federal de Goiás, Goiânia, GO, Brazil; 3 Instituto de Informática, Universidade Federal de Goiás, Goiânia, Goiás, Brazil

**Keywords:** *Paracoccidioides*, Antifungal, Oenothein B, Transcriptome, Cell wall

## Abstract

**Background:**

The compound oenothein B (OenB), which is isolated from the leaves of *Eugenia uniflora*, a Brazilian Cerrado plant, interferes with *Paracoccidioides* yeast cell morphology and inhibits 1,3-β-D-glucan synthase (*Pb*FKS1) transcript accumulation, which is involved in cell wall synthesis. In this work we examined the gene expression changes in *Paracoccidioides* yeast cells following OenB treatment in order to investigate the adaptive cellular responses to drug stress.

**Results:**

We constructed differential gene expression libraries using Representational Difference Analysis (RDA) of *Paracoccidioides* yeast cells treated with OenB for 90 and 180 min. Treatment for 90 min resulted in the identification of 463 up-regulated expressed sequences tags (ESTs) and 104 down-regulated ESTs. For the 180 min treatment 301 up-regulated ESTs and 143 down-regulated were identified. Genes involved in the cell wall biosynthesis, such as GLN1, KRE6 and FKS1, were found to be regulated by OenB. Infection experiments in macrophages corroborated the *in vitro* results. Fluorescence microscopy showed increased levels of chitin in cells treated with OenB. The carbohydrate polymer content of the cell wall of the fungus was also evaluated, and the results corroborated with the transcriptional data. Several other genes, such as those involved in a variety of important cellular processes (i.e., membrane maintenance, stress and virulence) were found to be up-regulated in response to OenB treatment.

**Conclusions:**

The exposure of *Paracoccidioides* to OenB resulted in a complex altered gene expression profile. Some of the changes may represent specific adaptive responses to this compound in this important pathogenic fungus.

## Background

Opportunistic and invasive fungal infections have become an important cause of morbidity and mortality [1]. The alarming rates of emerging antifungal resistance in hospitals are major concerns to the public health and scientific communities worldwide [2]. The most common antifungals currently used in the treatment of mycosis have some limitations, such as toxicity [3]. Considering all of these circumstances together has led to an urgent need for the identification of new and more effective antifungal agents.

Plants provide unlimited opportunities for the isolation of new antifungal compounds because of the unmatched availability of chemical diversity [4]. In fact, numerous antifungal compounds have been isolated from them [5-7]. The leaves of *Eugenia uniflora* L (Myrtaceae), known as the Brazilian cherry tree or pitangueira, are used in infusions or decoctions in popular medicine to treat inflammations associated with rheumatic pains and fever, hypoglycemia, as a diuretic and to prevent stomach diseases [8]. The hydroalcoholic extract from leaves decreased the levels of the enzyme xanthine oxidase, which is believed to be involved in the development of gout [9] in addition to its antioxidant and anti-inflammatory actions [10]. The enzyme also has anti-diarrheal [11], antihypertensive [12] actions and anti-microbial properties [13]. The Brazilian cherry tree is mentioned in the fourth edition of the Brazilian Pharmacopoeia [14], and the leaves are on the list of medicinal plants authorized by the Ministry of Health (ANVISA) with which to prepare infusions, as seen in the Brazilian Resolution number 267 [15].

Oenothein B (OenB) is a macrocyclic hydrolyzable tannin dimer isolated from *E. uniflora* leaves and possesses antimicrobial [16] and anti-tumor activities [17,18] and anti-inflammatory and anti-oxidant properties [10]. Bio-guided assays with leaves from *E. uniflora* showed that OenB inhibited *Paracoccidioides* growth and the accumulation of 1,3-β-D-glucan synthase (*Pb*FKS1) transcripts, which synthesizes 1,3-β-D-glucan polymer that is deposited in the fungus cell wall [19].

*Paracoccidioides* is a thermodimorphic fungus and is the etiologic agent of paracoccidioidomycosis (PCM), an important mycosis in Latin America. The infection is caused by the inhalation of the fungal spores, after which the fungus is able to disseminate from the lungs through the lymphatic system or the bloodstream to any organ or system [20]. The initial treatment of PCM depends on the severity of the disease and may last from 2 to 6 months. Extended periods of treatment, up to 2 or more years, are often necessary with a significant frequency of relapsing disease [21,22].

Several studies have been performed to investigate the transcriptional profile of the fungus in order to elucidate the mode of action of candidate drug compounds [23-26]*.* Here, cDNA libraries were constructed to obtain expressed sequences tags (ESTs) of *Paracoccidioides*. The Representational Difference Analysis (RDA) technique was used to identify changes in the transcriptional profile of *Paracoccidioides* in response to OenB, aiming to identify the probable mode of action of the compound in the fungus. Transcript levels were also measured during the infection process. In addition, the cell wall polymer levels were examined in members of the *Paracoccidioides* complex.

## Results

### Libraries characteristics

Cell viability was measured after exposure to OenB. A viability of 75% and 85% (data not shown) corresponding to the 90 and 180 min treatments, respectively, were chosen for RNA extraction and library construction. The libraries were constructed using the RDA technique, which is able to identify both up- and down-regulated differentially expressed genes between two cDNA populations [27]. The RDA experimental design included two conditions: (i) *Paracoccidioides* yeast cells exposed to McVeigh Morton (MMcM) medium with 0.32 M OenB for 90 and 180 min and (ii) *Paracoccidioides* yeast cells grown in MMcM medium for 90 and 180 min. For the up- regulated gene library construction, the first condition (i) was used as a tester population and the latter (ii) as the driver population. However, for the down-regulated library construction, the former (i) was used as the driver population and the latter (ii) as the tester population. Subtractions were performed by incubating the driver and tester populations. The selection of the cDNAs was achieved by constructing subtracted libraries in pGEM-T Easy, as described in the Methods section.

### Bioinformatics results for libraries exposed to OenB

RDA analysis revealed that a large number of ESTs (1011) was differentially regulated in the four gene libraries constructed after OenB treatment. From the RDA library of yeast cells exposed to OenB for 90 min 463 up-regulated and 104 down- regulated genes were identified. After pipeline analysis, the 463 up-regulated sequences originated 36 contigs and 20 singlets; the 104 down-regulated sequences originated 12 contigs and 7 singlets. The same analysis was performed for the RDA library obtained from yeast exposed to OenB for 180 min; 301 up-regulated and 143 down-regulated genes were identified from this library. After pipeline analysis, the 301 up-regulated sequences formed 14 contigs and 8 singlets; the 143 down-regulated sequences formed 12 contigs and 6 singlets. The ESTs obtained were submitted to the National Center for Biotechnology Information (NCBI) under accession numbers: LIBEST_028147 *Paracoccidioides* oenoteinB 90up Library, LIBEST_028146 *Paracoccidioides* oenoteinB 90 down Library, LIBEST_028149 *Paracoccidioides* oenoteinB 180 up Library and LIBEST_028148 *Paracoccidioides* oenoteinB 180down Library.

All of these up- and down-regulated ESTs after 90 and 180 min of exposure to OenB were compared to *Paracoccidioides* genes in the database of the Broad Institute by using the Blast X program. ESTs with e-value < 10^-5^ were considered in this analysis. All the obtained contigs and singlets were annotated by using the Blast2GO program. Based on this annotation, the ESTs represented in the four libraries were grouped into the functional categories according to the classification of GO. The ESTs were related with metabolism, energy, transcription, signal transduction, protein fate, protein synthesis, morphology, transport, cell cycle and DNA processing, cell rescue, defense and virulence, functionally unclassified proteins and unclassified proteins (Table 1).

**Table 1:** **Annotated ESTs up and down regulated genes of *Paracoccidioides *yeast cells treated with OenB by 90 and 180 min**

**Functional category**	**Gene product**	***Paracoccidioides *acession number**^**a**^	**e-value**	**Number of occurrence**	
				**90 min**	**180 min**
**Metabolism**					
**Amino acids metabolism**	Alanine glyoxylate aminotransferase **(AGX1)**	PAAG_03138.1	1e-152	+2	-1
	Hexokinase 1 **(HXK1)**	PAAG_01377.1	2e-48	+2	
	Urease **(URE)**	PAAG_00954.1	0.0	+1	
**Carbohydrate metabolism**	Betaine aldehyde dehydrogenase **(BADH)**	PAAG_05392.1	0.0	-13	-16
	β glucosidase **(BGLU)**	PAAG_04545.1	0.0	+1	
	Cysteine desulfurase **(NFS1)**	PAAG_05850.1	2e-122	+4	-7
	Trehalose phosphatase **(TPS1)**	PAAG_06703.1	1e-165	+20	
**Fatty acid metabolism**	C-5 sterol desaturase **(ERG 3)**	PAAG_03651.1	1e-166	-4	
	NADP-dependent leukotriene B4 12-hydroxydrogenase **(LTB**_**4**_**DH)**	PAAG_05416.1	5e-179	+1	
	Fatty acid elongase **(GNS1/SUR4)**	PAAG_08553.1	0.0	+2	
** Polysaccharide biosynthesis**	Glutamine synthetase **(GLN1)**	PAAG_07003.1	0.0	+11	+1
**Energy**					
**Fatty acid β-oxidation**	Acyl-CoA dehydrogenase **(ACAD)**	PAAG_03490.1	2e-85	+1	
**Energy generation**	Glucose methanol choline oxidorreductase **(GMCO)**	PAAG_08146.1	0.0		+3
**Morphology**	β glucan synthesis associated protein **(KRE6)**	PAAG_00091.1	2e-36		+3
	α 1,6 mannosyltransferase **(OCH1)**	PAAG_01658.1	0.0	+5	
	Arp 2/3 complex subunit Arc16	PAAG_03624.1	0.0		+12
	Cell morphology protein **(PAL1)**	PAAG_02031.1	0.0	+1	
	Integral membrane protein **(MPV17/PMP22)**	PAAG_02868.1	4e-87	-6	
	GYF domain protein	PAAG_00627.1	0.0	+5	
**Transcription**	C_2_H_2_ transcription factor **(SEB1)**	PAAG_03287.1	0.0	+2	
	C6 transcription factor **(CTF1B)**	PAAG_01359.1	0.0	-15	+54
	Fork head box protein D1 **(FOXD1)**	PAAG_07388.1	4e-40	+1	
	GATA type sexual **(NSDD)**	PAAG_05818.1	3e-127	+22	-4
	Histone deacetylase **(RPD3)**	PAAG_06742.1	0.0	+12	
	RING finger protein **(RNF)**	PAAG_06129.1	0.0	-3	-15
	Transcription factor **(ATF1)**	PAAG_01945.1	5e-169	+1	
	Transcription factor fungi	PAAG_02049.1	0.0		+65
	Protein RNP domain	PAAG_03136.1	6e-98	+2	
	Transcription factor prr1**(HSF1)**	PAAG_05064.1	6e-89	+1	
	Transcription factor **(STEA)**	PAAG_00406.1	0.0	+18	
**Signal transducer**	Guanine nucleotide binding protein alpha-1 subunit **(GPA2)**	PAAG_04436.1	5e-178	-23	
	Protein with PYP-likesensor domain **(PAS)**	PAAG_06301.1	0.0	+10	-44
**Protein fate**	Proteasome component **(PRE6)**	PAAG_07802.1	7e-70		+3
**Protein synthesis**	Elongation factor 1-gamma 1 **(eEF-1)**	PAAG_03556.1	0.0		-1
	ATP-dependent RNA helicase **(eIF-4A)**	PAAG_00689.1	0.0	+2	-9
**Cellular transport, transport facilities and transport routes**	Calcium-transporting ATPase sarcoplasmic/endoplamic reticulum type **(PMR1)**	PAAG_00774.1	0.0	+1	
	Ferric-chelate reductase **(FRE)**	PAAG_05370.1	7e-107		+2
	Succinate/fumarate mitochondrial transporter **(SFC1)**	PAAG_06563.1	0.0	-4	
	Membrane biogenesis protein **(YOP1)**	PAAG_00481.1	7e-143		+1
	Major facilitator superfamily transporter **(MFS)**	PAAG_01353.1	0.0	-27	-22
	Bodown 198 – Major facilitador superfamily transporter	PAAG_06077.1	3e-124	-4	
**Cell cycle and DNA processing**	Ribonuclease reductase large subunit **(RNR1)**	PAAG_02210.1	5e-175	+1	
	Arginine N-methyltransferase - SKB1 **(PRMT5)**	PAAG_02402.1	0.0	+52	
	SH3 domain-containing protein **(CYK3)**	PAAG_02301.1	0.0	+2	
**Cell rescue, defense and virulence**					
**Stress Response**	Phosphatase regulatory subunit **(GAC1)**	PAAG_00128.1	0.0	+10	
	Heat shock protein **(HSP70)**	PAAG_08003.1	1e-74	-1	
**Virulence factor**	Pathogenesis associated protein **(CAP20)**	PAAG_06538.1	0.0		-21
**Functional unclassified proteins**	Uncharacterized protein family UPF0121	PAAG_00184.1	9e-94		+4
	Pleckstrin Homology (PH) domain	PAAG_03092.1	0.0	+11	
	DUF 1688 domain protein	PAAG_04190.1	0.0	+154	
**Unclassified proteins**	Conserved hypothetical protein	PAAG_06834.1	0.0	+25	
	Conserved hypothetical protein	PAAG_07365.1	0.0	+34	
	Conserved hypothetical protein	PAAG_05009.1	0.0	+10	-3
	Conserved hypothetical protein	PAAG_07364.1	2e-139	+4	+2
	Conserved hypothetical protein	PAAG_04732.1	0.0	+7	
	Conserved hypothetical protein	PAAG_03559.1	0.0	+2	
	Conserved hypothetical protein	PAAG_02868.1	4e-87	+11	
	Conserved hypothetical protein	PAAG_01170.1	9e-122	+1	
	Conserved hypothetical protein	PAAG_04190.1	0.0	-1	
	Conserved hypothetical protein	PAAG_06925.1	0.0		+1
	Conserved hypothetical protein	PAAG_08832.1	3e-143		+5
	Conserved hypothetical protein	PAAG_07770.1	3e-143		+1
	Conserved hypothetical protein	PAAG_03654.1	2e-70		+63
	Conserved hypothetical protein	PAAG_00520.1	1e-7		+1
	Hypothetical protein	PAAG_07199.1	7e-126		+7
	Hypothetical protein	PAAG_03580.1	0.0	+6	
	Hypothetical protein	PAAG_04733.1	0.0	+3	
	Hypothetical protein	PAAG_06820.1	0.0	+1	
	Hypothetical protein	PAAG_08066.1	7e-71		+27
	Hypothetical protein	PAAG_05558.1	7e-76		+1
	Hypothetical protein	PAAG_03580.1	0.0		-1
	Hypothetical protein	PAAG_03099.1	2e-151		+45
	Hypothetical protein	PAAG_06251.1	0.0	-3	
	Hypothetical protein	PAAG_01169.1	0.0	+1	
	Hypothetical protein	PAAG_08809.1	0.0		+1

^a^ Accession number at Broad (http://www.broadinstitute.org).

Graphs were plotted to demonstrate the statistically enriched GO functions with up- or down-regulated genes after exposure to the compound (Additional file 1: Figure S1). The groups with the highest percentage of up-regulated genes were the unclassified proteins (30%), transcription (23%), functional unclassified proteins (22%), metabolism (10%), cell cycle and DNA processing (7%) and morphology (4%). The highest percentage of down-regulated genes was grouped in the signal transducer (27%), cellular transport, transport facilities and transport routes (22%), transcription (15%), cell rescue, defense and virulence (14%), metabolism (12%), unclassified proteins (4%), protein synthesis (4%) and morphology (2%) groups.

**Figure 1:**
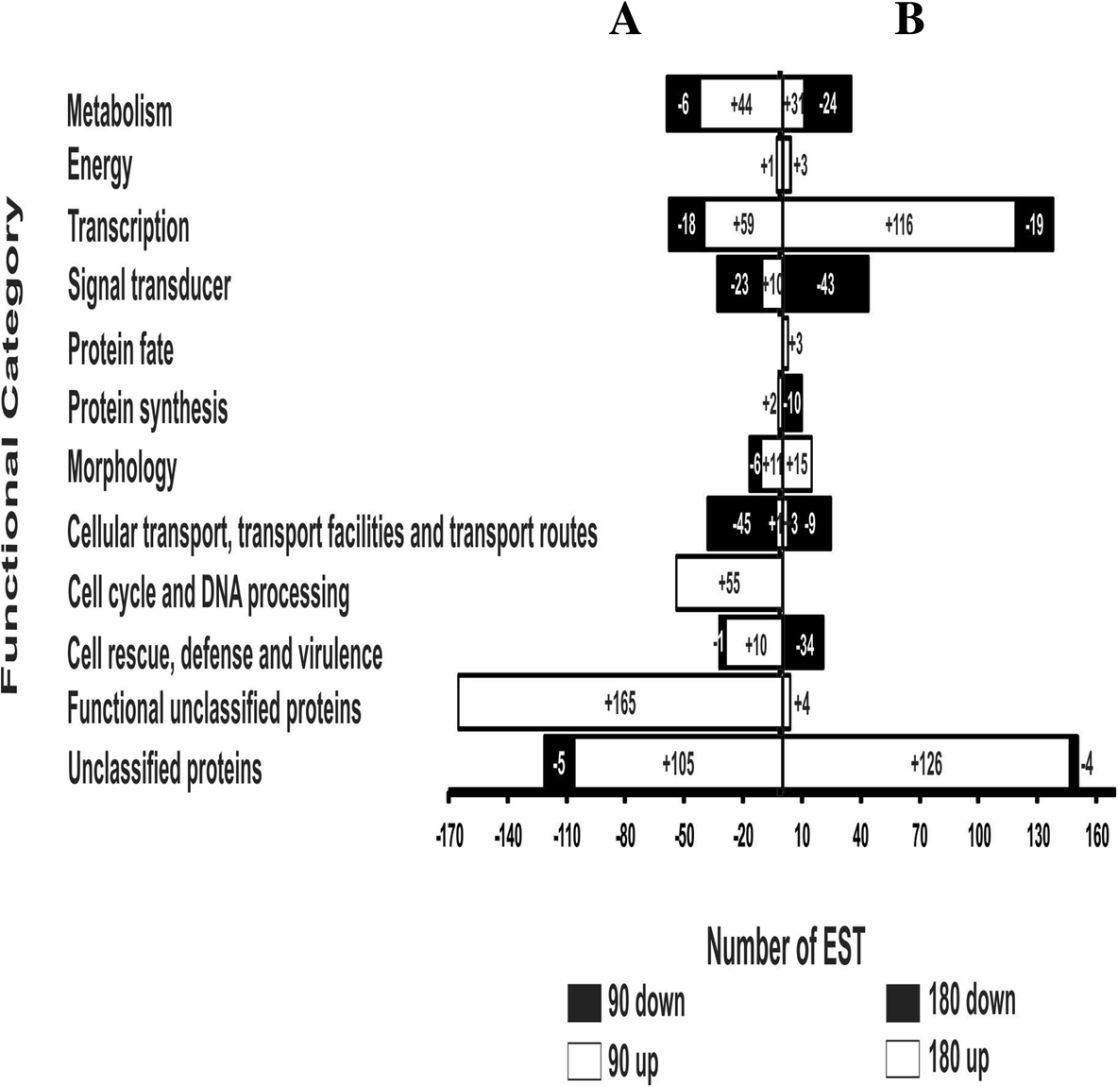
**Functional classification of differentially expressed genes in biological functional groups. (A)** Down- and up-regulated genes obtained from cDNAs synthesized from RNAs of *Paracoccidioides* cells exposure to OenB for 90 min and **(B)** 180 min. Functional classification was based on Blast X homology of each EST against the GenBank non-redundant database and the nucleotide database from the *Paracoccidioides* structural genome at a significant homology cut-off ≤ 10^-5^ and a MIPS functional annotation scheme. Each functional class is represented as a segment and expressed as the total number of ESTs in each library.

The distribution of differentially expressed genes in the different functional categories and biological processes was evaluated (Table 1; Figure 1). The analysis demonstrated that most of the genes that were down-regulated after 90 min participate in cellular processes related to transport, followed by signal transducers and those involved in transcription and metabolism. On the other hand, the up-regulated genes were mostly found to be functionally unclassified proteins, followed by unclassified proteins, and those involved in cell cycle and DNA processing and metabolism. After 180 min treatment, most of the down-regulated genes were also involved in signal transduction, metabolism and cellular transport. The up-regulated genes were unclassified proteins or those involved in transcription and morphology determination. The percentage of occurrence of each gene in the libraries was indicated in the Additional file 2: Table S1.

**Figure 2:**
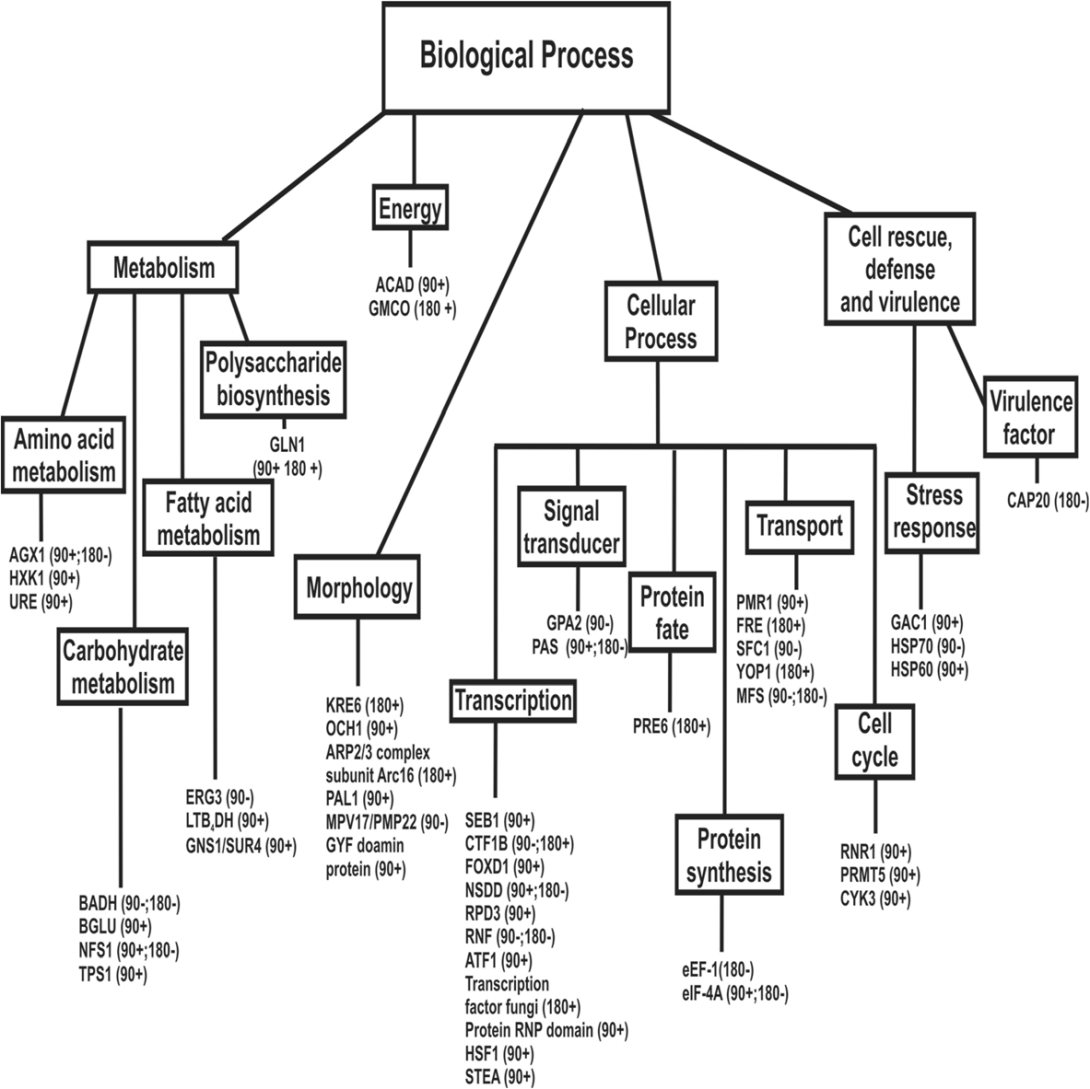
**Distribution of genes responding to OenB in *Paracoccidioides*.** The most evident up- and down-regulated genes are shown. Data were organized into various biological processes using Functional Categories MIPS and GO tools. A positive signal indicates increased, and negative values indicate reduced transcript levels. A complete list of all significant genes can be found in Table 1.

The OenB treatment resulted in up-and down-regulated genes involved in different biological processes (Table 1; Figure 2). We analyzed the occurrence of the transcripts by determining the number of ESTs found in each transcript. The highest occurrence of up-regulated ESTs after 90 min of OenB treatment were as follows: DUF 1688 domain protein (154 ESTs), arginine N-methyltransferase- SKB1 (PRMT5) (52 ESTs), GATA-type sexual (NSDD) (22 ESTs), trehalose phosphatase (TPS1) (20 ESTs), histone deacetylase (RPD3) (12 ESTs), glutamine synthetase (GLN1) (11 ESTs), phosphatase regulatory subunit (GAC1) (10 ESTs) and protein with a PYP-like sensor domain (PAS) (10 ESTs). For down-regulated ESTs, the highest expression transcripts were the following: major facilitator superfamily transporter (MFS) (27 ESTs), guanine nucleotide binding protein alpha-1 subunit (GPA2) (23 ESTs) and C6 transcription factor (CTF1B) (15 ESTs).

Likewise, we analyzed the up-and down-regulated genes resulting from 180 min of OenB treatment and noted genes that might be involved in different biological process (Table 1; Figure 2). The highest transcription expression of transcripts after 180 min of treatment were the following for up-regulated ESTs: transcription factor fungi (65 ESTs), glutamine synthetase (GLN1) (1 EST) and arp 2/3 complex subunit Arc16 (12 ESTs). For down-regulated ESTs, the highest transcript expression of transcripts were the following: protein with PYP-like sensor domain (PAS) (44 ESTs), pathogenesis associated protein (CAP20) (21 ESTs), betaine aldehyde dehydrogenase (BADH) (13 ESTs) and RING finger protein (RNF) (15 ESTs). The most evident up- and down-regulated genes are shown in the Figure 2.

### Quantitative real-time PCR (qRT-PCR)

RDA is a methodology that enriches for differentially expressed sequences within transcriptional profiles and specifically permits the detection of transcripts that are differentially represented even at low levels of expression, such as transcription factors and other regulatory genes. While representative in general terms, further confirmation of identified sequences is indicated, as in any high throughput approach, and qRT-PCR is a method of choice for this confirmation process.

Consequently, we designed gene-specific primers for a set of the ESTs obtained from the RDA libraries of *Paracoccidioides* yeast cells treated with OenB for 90 and 180 min. In this set, we evaluated genes represented in the libraries by one or more ESTs and to genes from a different functional GO category. For all seven selected genes, we obtained primer sequences that amplified specific products. Some genes selected for confirmation by qRT-PCR were also assessed after 24 h of infection of macrophages by *Paracoccidioides*. These sequences are shown in Table 2.

**Table 2:** Oligonucleotide primers used in RDA assay and qRT-PCR

**Sequence name**	**Forward primer (5′- 3′)**	**Reverse primer (5′- 3′)**	**Reaction**
cDNA	AGCAGTGGTATCAACGACAGAGTACGCGGG		Synthesis of the first-strand for RDA
CDS	AAGCAGTGGTATCAACGCAGAGTACT(30)N1N		Synthesis of the first-strand for RDA
PCRII	AAGCAGTGGTATCAACGCAGAGT		Synthesis of the first-strand for RDA
JBam12, 24	GATCCGTTCATG	ACCGACGTCGACTATCCATGAACG	Adapter 1 (RDA)
NBam12,24	GATCCTCCCTCG	AGGCAACTGTGCTATCCGAGGGAG	Adapter 2 (RDA)
T7	GTAATACGACTCACTATAGGGC		Sequencing
Oligo (dT)_15_	AAGCAGTGGTATCAACGCAGAGTACT(30)N1N3′		Synthesis of the first-strand for qRT-PCR
CAP20	CCTTCACGAACTCGCCACTAT	TCGCTGCTTAGGGAGTCTGC	qRT-PCR
FKS1	GACAACAGAGGGTATAATGGG	GCCATATTGATAGCCTGCAGC	qRT-PCR
GLN1	CGATCAAAAACAAAGACCCT	GGTCTGGGTACATGGCAAC	qRT-PCR
KRE6	GGTATATGCCTAACTTTGAATTC	GCGTAGACTTGATACTCTTTTG	qRT-PCR
GAC1	AGTACTGCTTCTATGGATCTTC	ACTATTTCCTGGGGTCGTTG	qRT-PCR
MFS	CTAATTATGTTCTTTTGGGGTAC	GCATCGCCTATACCAACAAGA	qRT-PCR
PAL1	TGCTGCGGAACTCTTTGA	GGGCTTATCGTCGGAGAGTC	qRT-PCR
ERG3	CACTTGGATCTTCGGCCTAAT	TGCATAGCCACGGACTTCGA	qRT-PCR

(N1 = A, G or C/N = A, C, G or T).

**Figure 3:**
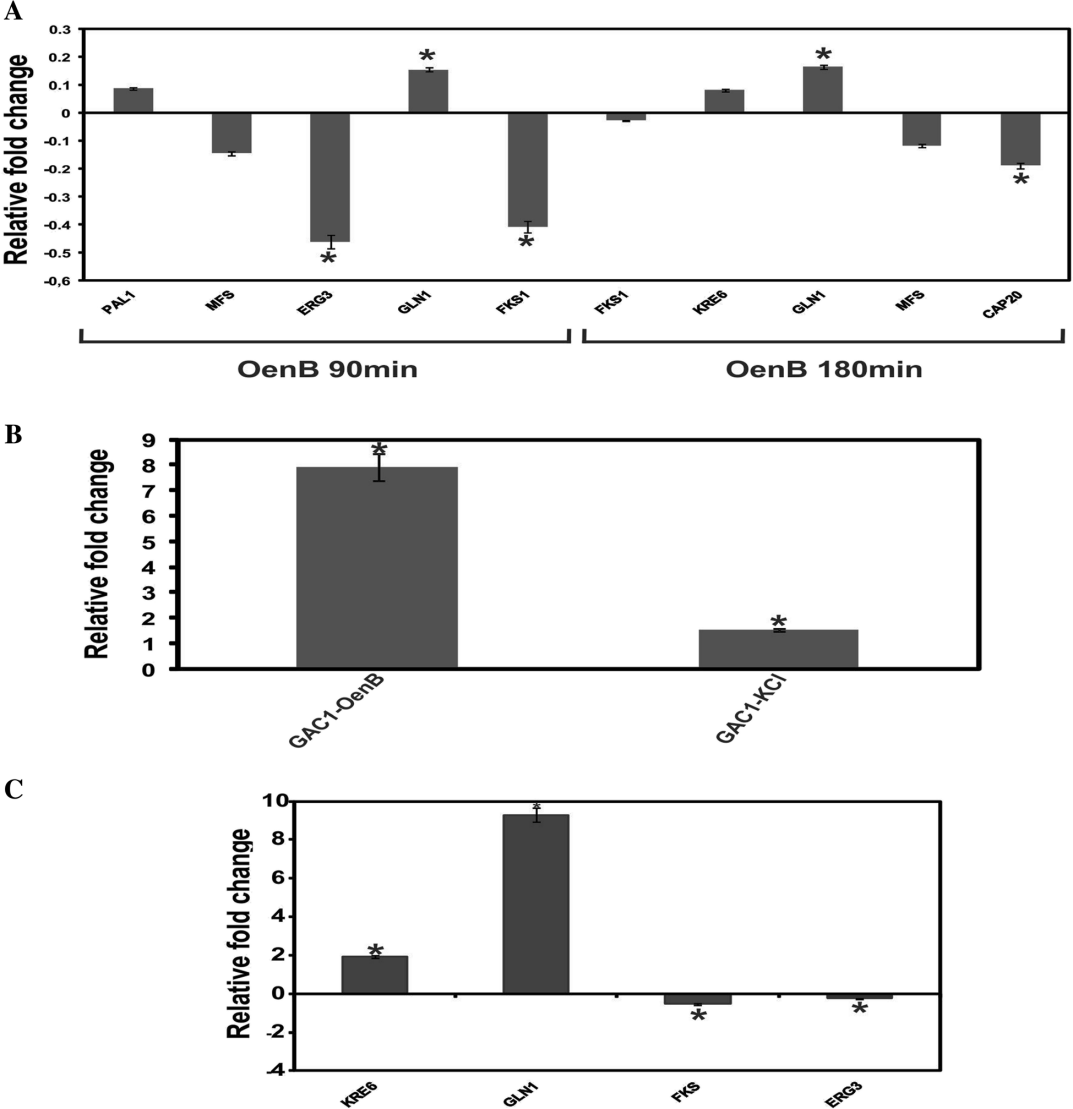
**Relative fold change for genes PAL1, MFS, ERG3, GLN1, FKS1, KRE6, CAP20 and GAC1. (A)** Gene expression profile of *Paracoccidioides* yeast cells exposed to OenB after 90 and 180 min. **(B)** GAC1 gene expression of *Paracoccidioides* yeast cells exposed to a causative agent osmotic stress, KCl. **(C)** Gene expression profile of *Paracoccidioides* yeast cells used to infect macrophage cells exposed to OenB after 24 h. Changes in gene expression levels were calculated by the relative standard curve method using the non-treated control samples as the calibrator. Each error bar represents the standard error of the mean (±SD), and significant-fold changes are denoted by asterisks in the figure (*p ≤ 0.05). Data were normalized with the transcript encoding the α-tubulin protein.

The expression levels of the up- and down-regulated genes from the library of yeast treated for 90 min were confirmed as significantly higher for PAL1 and GLN1 and significantly lower for the MFS and ERG3 genes, compared to the controls, which was in accordance with the results from the RDA methodology. All four genes from the library of yeast treatment for 180 min also corroborated the results from the RDA methodology because the levels of expression were significantly higher for GLN1 and KRE6 and significantly lower for MFS and CAP20 genes (Figure 3A).

The FKS1 gene was analyzed because we found in our previous work that the gene was repressed in the presence of OenB [19]. Here, the decreased level of FKS1 after 90 and 180 min of treatment are in agreement with our previous data.

Previous studies using electron microscopy have demonstrated the formation of large vacuoles with almost no electron density, the presence of lysed cells, partial disruption of the cell wall and cytoplasmic organelle leakage out of OenoB-treated *Paracoccidioides* yeast cells [19]. Here, genes related to osmotic stress, such as RPD3, ATF1, HSF1 and GAC1 were up-regulated in *Paracoccidioides* yeast cells after OenB-treatment. In this way, the GAC1 gene, which responds to osmotic stress [28], was evaluated in the presence of KCl. It is noteworthy that KCl was used in this study as a stressor because it is an osmotic stress inducer and we found that it acted on the cell wall of *Paracoccidioides* in our previous study [29]. Our results indicated that the GAC1 gene responds to osmotic stress because its expression level was increased in the presence of OenB and KCl (Figure 3B).

### Transcript levels in OenB-treated *Paracoccidioides* yeast cells after internalization by macrophage cells

To investigate if the transcripts identified by RDA would also be up- or down- regulated *in vivo*, *Paracoccidioides* yeast cells were internalized by macrophage cells and the transcript levels were then quantified. J774A.1 mouse macrophage cells infected with *Paracoccidioides* were treated with OenB for 24 h, and qRT-PCR analysis was carried out for the genes KRE6, GLN1, FKS and ERG3. The expression levels of genes found to be up- and down-regulated in the libraries of yeast cells treated for 90 and 180 min were confirmed. Expression was significantly lower for ERG3 and significantly higher for KRE6 and GLN1 after 24 h of infection. The FKS1 gene, which was down- regulated in the presence of OenB in our previous work [19], was also dow- regulated during macrophage infection (Figure 3C). The results are in agreement with those found in the RDA and qRT-PCR assays when *Paracoccidioides* yeast cells were treated with OenB *in vitro*.

### Cell analysis by fluorescence microscopy

Calcofluor White (CFW) and Congo Red (CR) both stain the cell wall of fungi by binding to chitin chains [30]. CFW and CR dyes interact with various polysaccharides [31] but exhibit a particularly high affinity for chitin [32,33].

**Figure 4:**
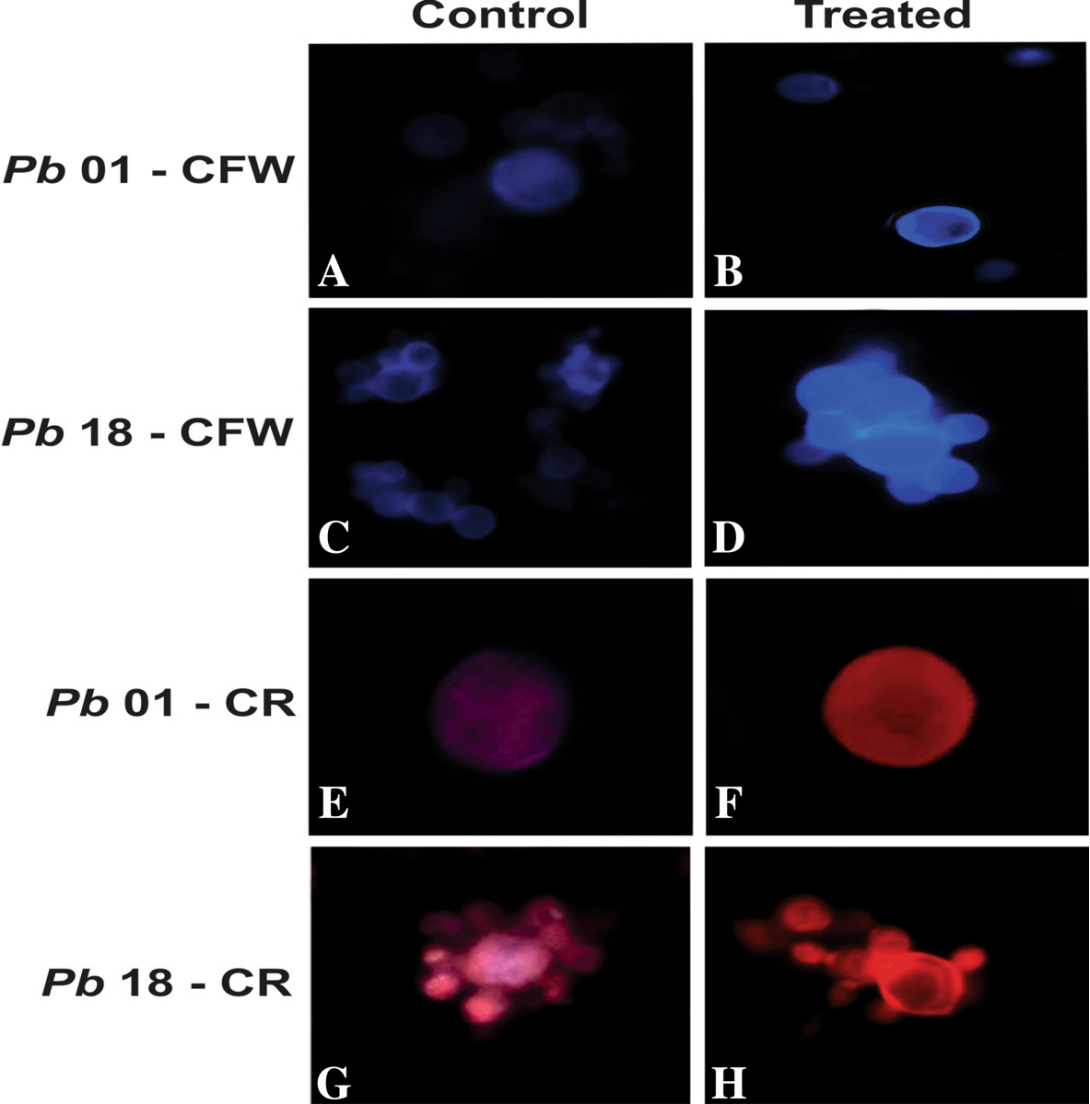
**Fluorescence microscopy showing *Paracoccidioides Pb*01 and *Pb*18 isolates stained by CFW and CR. (A) ***Pb*01 control stained with CFW; **(B) ***Pb*01 after treatment with OenB, stained with CFW; **(C) ***Pb*18 control stained with CFW; **(D) ***Pb*18 after treatment with OenB, stained with CFW; **(E) ***Pb*01 control stained with CR; **(F) ***Pb*01 after treatment with OenB, stained with CR; **(G) ***Pb*18 control stained with CR; **(H) ***Pb*18 after treatment with OenB, stained with CR. The cells after treatment with OenB showed intense fluorescence when stained with CFW and CR.

To investigate the chitin level in the cell wall, *Paracoccidioides Pb*01 and *Pb*18 yeast cells were grown in liquid medium for 72 h with and without OenB, after which they were stained with Calcofluor White (CFW) and Congo Red (CR) and visualized by fluorescence microscopy. OenB-treated *Paracoccidioides* cells showed a stronger fluorescence when compared to the control, indicating increased chitin levels (Figure 4).

### Carbohydrate content of the cell wall

**Figure 5:**
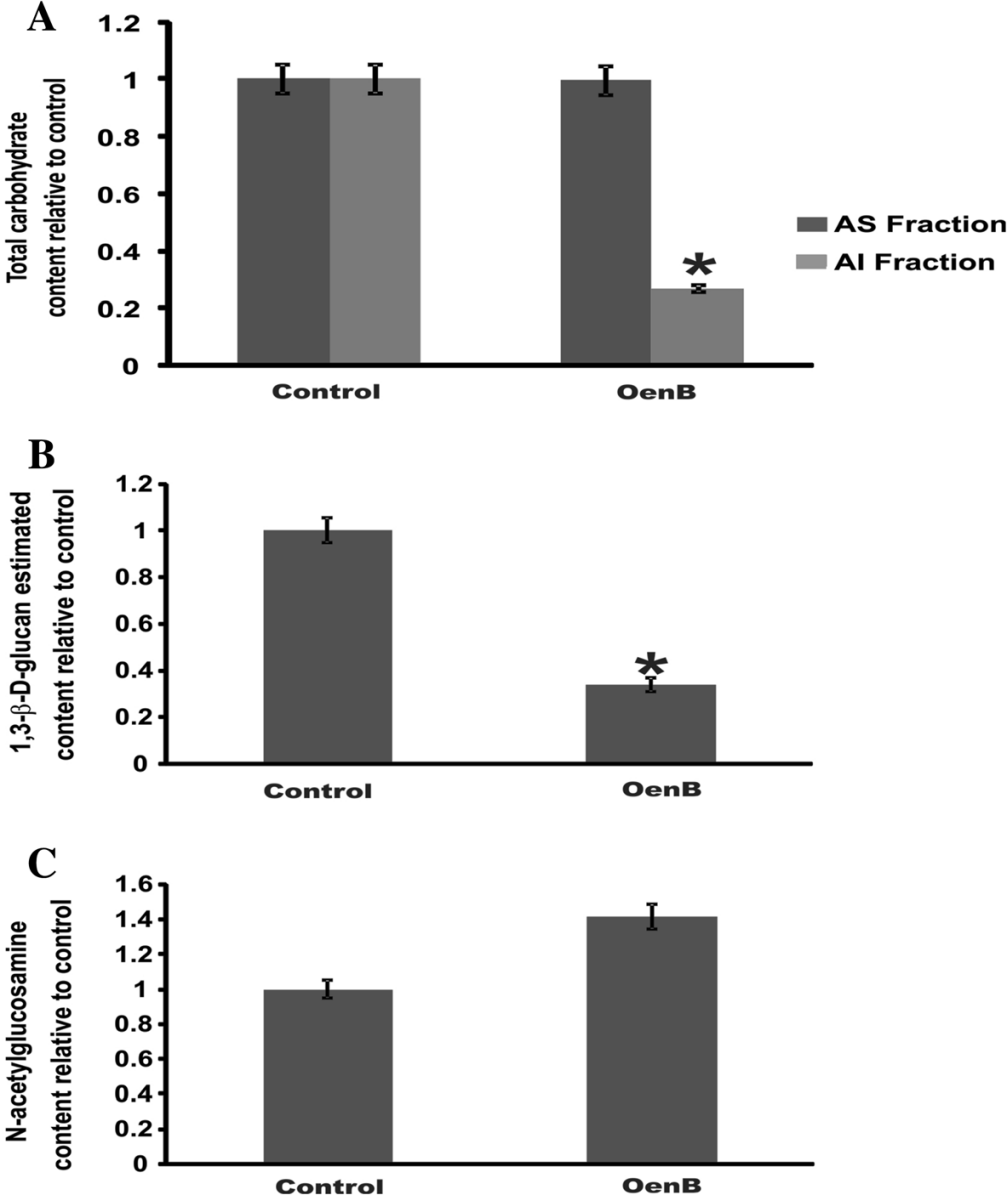
**Carbohydrate content in the cell wall of *Paracoccidioides *yeast cells exposed to OenB.** The cell walls of *Paracoccidioides* that were either untreated (control) or treated with OenB were isolated. The alkali-soluble (AS) and alkali-insoluble (AI) fractions were separated. **(A)** Total amount of carbohydrates was measured in the AS and AI fractions. **(B)** The amount of 1,3-β-D-glucan polymer was estimated in the AI fraction. **(C)** The amount of N-acetylglucosamine (GlcNAc) residue was measured in the total cell wall fraction. All data were normalized relative to the control. Three independent experiments were performed. *Significantly increased amount (*p* ≤ 0.05).

The cell wall is divided in alkali-soluble (AS) and alkali-insoluble (AI) fractions according to its solubility in alkaline substances. In the *Paracoccidioides Pb01* yeast phase, the glucan polymer consists mainly of 1,3-α-glucan (95%) which is present in the AS fraction, and a small amount of the 1,3-β-D-glucan (5%), which is present in the AI fraction [34]. GlcNAc residues are also found in the AI fraction. Figure 5A shows a decrease in total carbohydrate content in the AI fraction after OenB-treatment; no alteration in carbohydrate content was observed in the AS fraction. 1,3-β-D-glucan content was estimated after the digestion of the AI cell wall fraction with the 1,3-β-glucanase enzyme. As shown in Figure 5B, there was a decrease in 1,3-β-D-glucan content. In addition there was an increase in GlcNAc residue content in this fraction (Figure 5C), suggesting an increase in the chitin polymer concentration.

## Discussion

The aim of this study was to identify changes in the gene expression profile of *Paracoccidioides* yeast cells after OenB treatment in order to investigate the adaptive cellular responses of the fungus. In the RDA libraries, genes related to metabolism, energy, morphology, transcription, transport, among others were found to be differentially expressed relative to the control.

In our previous work, were visualized several events indicative of *Paracoccidioides* cell wall collapse in the presence of OenB [19]. In the cDNA libraries, some genes involved in the biosynthesis of the polysaccharide cell wall (GLN1) and morphology (KRE6, OCH1 and MPV17/PMP22) were up-regulated in OenB-treated *Paracoccidioides* yeast cells. Although the FKS1 transcript was not found in the cDNA library, the down-regulation of the FKS1 transcript was confirmed by qRT-PCR, thus corroborating previous data [19]. The decreased amount of the 1,3-β-D-glucan polymer was confirmed here after the digestion of the cell wall.

The cell wall composition of *Paracoccidioides* varies biochemically depending on its phase. The cell wall of the yeast phase, found in infected tissue and *in vitro* culture at 36°C [35,36], has more chitin polymer and less 1,3-β-D-glucan polymer than found in the saprophytic mycelium phase. The 1,3-α-D-glucan polymer was only found in the yeast phase [34,37]. The polysaccharide α-glucan is supposedly located in a more external region of the cell wall and is related to virulence [38,39]. Both 1,3-β-D-glucan and chitin are linked to cell wall structural function. In addition, 1,3-β-D-glucan is an immunogenic molecule [39,40].

Glutamine synthetase (GLN1) was up-regulated in OenB-treated *Paracoccidioides* yeast. GLN1 catalyzes the synthesis of glutamine from glutamate and ammonia [41] and contributes glutamine to the synthesis of chitin [42]. Here, the increase of chitin level was confirmed by fluorescence microscopy. In *Mycobacterium bovis*, GLN1 is necessary for cell wall resistance [43]. In previous studies conducted with *Paracoccidioides*, the increase in GLN1 transcript was related to chitin deposition in response to change in external osmolarity faced by the fungus [44]. The increase in chitin synthase transcripts and chitin synthesis in *C. albicans* and *A. fumigatus* after treatment with antifungal agents has been shown to be an important compensatory response to cell wall stress [45,46].

The increase in chitin synthesis has been shown to be an important compensatory response to cell wall stress. In *C. albicans*, increased chitin synthase gene expression has been observed after treatment with micafungin, implying that the induction of chitin synthesis might contribute to cell survival when the cell wall is damaged [45]. In addition the transcription levels of *chsA* and *chsC*, two chitin synthases, were increased in *A. fumigatus* after treatment with echinocandin [46].

β-glucan synthesis associated protein (KRE6) was also up-regulated in *Paracoccidioides* after 180 min of OenB-treatment. KRE and KRE-related genes are required for the synthesis of cell wall 1,6-β-glucan in *S. cerevisiae*[47]. In addition, the disruption of *kre6* reduces 1,6-β-glucan levels [48]. The 1,6-α-glucan and the genes involved in its synthesis are critical for maintaining the growth, morphology and cell wall integrity of *C. neoformans*[49].

The 1,6-α-mannosyltransferase (OCH1) gene was up-regulated in *Paracoccidioides* after 90 min of OenB-treatment. OCH1 has many functions, including its inclusion in protein structures, its involvement in the maintenance and morphology of the cell wall and cell membrane, the synthesis of the extracellular matrix, storage of nutrients, and can also function as a ligand for cell-cell interaction [50]. In *Neurospora crassa*, OCH1 is required for the incorporation of cell wall proteins into the cell wall matrix [51]. In yeast including *S. cerevisiae, C. albicans* and *Candida glabrata*, mannan polymer (mannoprotein) provides the strength and shape for the resistance to environmental stress [52]. OCH1 transcription, which is regulated by the transcription factors ATF1, HSF1, and RPD3 cited above [53], may synthesize mannoproteins to be deposited in the cell wall [54].

Mannoproteins, chitin and β-1,6-glucan are required to maintain the cell wall and are therefore essential to cell viability [55]. Here, several genes involved in cell wall biosynthesis were up-regulated. The fungal cell wall is an important organelle that protects the cell from various environmental stressors. It is a dynamic structure that interacts with the environment and is modified to accommodate growth, cell division, and development [56,57]. In our previous work using electronic microscopy, several hallmark changes were found, including squashing, the presence of vacuoles with low electron density, a rough surface, cell wall rupture and cell membrane recess [19].

Others genes related to cellular morphology were also shown to be differentially regulated, such as ERG3 and MPV17/PMP22. The C-5 sterol desaturase (ERG3) gene was down-regulated in *Paracoccidioides* yeast treated with OenB for 90 min. ERG3 acts in the ergosterol biosynthesis, which is one of the most important components of fungal membrane [58]. It maintains the integrity and fluidity of the cell membrane [59] and consequently may affect the morphology [60], virulence [61] and resistance of fungal pathogens [62]; its biosynthetic pathway is essential for fungal growth [63]. The presence of cell membrane recess and folding to the inner cytoplasm initiating the formation of vacuoles was observed in OenB-treated *Paracoccidioides* yeast cells [19].

The transmembrane protein (MPV17/PMP22) was down-regulated in *Paracoccidioides* after 90 min of OenB treatment. In *S. cerevisiae*, *Sym1*, which is homologous to MPV17, is involved in the structural and functional stability of the inner mitochondrial membrane, thus controlling crucial mechanisms related to this compartment, including the activity of respiratory chain complexes, the mitochondrial morphology and the maintenance and integrity of nucleotide structures [64]. The leakage of cytoplasmic organelles was observed in *Paracoccidioides* yeast cells that were treated with OenB [19].

The major facilitator superfamily transporter (MFS) gene was down-regulated in OenB-treated *Paracoccidioides* yeast. The MFS transporters include important families of membrane protein transporters which utilize diverse substrates and catalyze different modes of transport [65]. In *S. cerevisiae* and *Candida spp.*, the up-regulation of multidrug transporter genes belonging either to the ABC family or to the MFS family is frequently observed in cells exposed to drugs and leads to the phenomenon of MDR (multidrug resistance) [65,66]. However, there are compounds, such as FK506, enniatins, milbemycins, synthetic D-octapeptides, cyclosporine, isonitrile, disulfiram, ibuprofen, and unnarmicins, that inhibit fungal ABC transporters [67,68]. Such inhibitors or chemo sensitizers probably act directly by affecting substrate binding and transport mediated by MDR efflux proteins [69]. In *S. cerevisiae*, the natural product curcumin competitively inhibitis R6G (rhodamine 6G), an MFS transporter [69]. Clorgyline, a drug previously used as an antidepressant, was found to enhance the antifungal effect of fluconazole against azole-resistant yeast species, including *C. albicans* and *C. glabrata* strains, via the inhibition of ABC and MFS fungal efflux pumps [70].

Although echinocandin, an antifungal acting at the fungal cell wall, provides fungicidal activity against *Candida spp.*, it is only fungistatic against *Aspergillus spp.*[71]. In *Paracoccidioides*, micafungin and echinocandin are more active against mycelia than against the yeast phase [72], although caspofungin can partially inhibit yeast phase growth, in different *Paracoccidioides* isolates [73]. In addition, it is important to develop antifungals that are active against the yeast phase because that is the pathogenic phase [74]. Our data indicate that OenB interferes with the *Paracoccidioides* cell wall. Because this structure is found in the fungus but not in human beings, it could be nontoxic to humans and may be an interesting antifungal prototype candidate.

## Conclusions

Our results indicate that the exposure of *Paracoccidioides* to OenB results in a complex altered gene expression profile. Some of the changes might represent specific adaptive responses to this compound because genes involved in the cell wall were differentially expressed, and polymer and image analysis fluorescence microscopy corroborated with transcriptome data.

## Methods

### Plant material

*E. uniflora* leaves were collected in Anápolis, Goiás, Brazil and the sample of specimens were deposited at the Herbarium of the Universidade Federal de Goiás UFG, with code number UFG 25477. Access authorization and sample remittance of component of the genetic property is n°005/2007.

### Extraction, isolation and identification of OenB from *E. uniflora*

The extraction, isolation and identification of OenB were realized as described previously [19]. Briefly, powdered leaves of *E. uniflora* (1.0 kg) were extracted with 50% aqueous acetone. The dry crude extract (174.27 g) was obtained by concentration in vacuum followed by freeze-drying and a part (150 g) was extracted with ethyl acetate. The aqueous layer was lyophilized to yield a 122.14 g fraction which was dissolved in methanol (MeOH) to separate the soluble (81.76 g) and the insoluble (39.22 g) methanolic fractions. The insoluble fraction (10 g) was submitted to separation and purification by Column Cromatography on Diaion HP-20, followed by CC on Sephadex LH-20, eluted with a water-MeOH gradient to obtain the IM-7 sub-fraction (791.5 mg). From this, OenB (62.4 mg) was isolated (purity of 96%) on a preparative HPLC system. The chromatographic system consisted of LC-10ADvp pumps (Shimadzu Corporation, Tokyo, Japan) connected to an SPD-10AVvp ultraviolet photodiode array detector. Chromatographic separations were performed using a LiChrospher RP-18 column (Shimadzu Corporation). The compounds were eluted using acetonitrile (A) 0.01 M H_3_PO_4_: 0.01 M KH_2_PO_4_ (B) (gradient 8% A in B to 18%) for 20 min at a flow rate of 1.0 mL/min.

### *Paracoccidioides* culture and determination of cell viability

*Paracoccidioides* (ATCC MYA 826) has been studied extensively in our laboratory [75-78]. *Paracoccidioides Pb*01 yeast cells were sub-cultured every seven days on solid Fava-Netto’s medium (1.0% w/v peptone, 0.5% w/v yeast extract, 0.3% w/v protease peptone, 0.5% beef extract, 0.5% w/v NaCl, 4% w/v glucose and 1.4% w/v agar, pH 7.2) [79] at 36°C and used throughout this study. For viability experiments, yeast cells were grown in the presence or absence of OenB for specific time intervals and were kept in MMcM chemically defined medium [80] at 36°C. The cellular viability was determined by the trypan blue method [81]. The quantity of OenB used was 500 μg/mL (0.32 M), which corresponds to minimal inhibitory concentration (MIC) [19]. In brief, cells were incubated with the dye solution (0.1% trypan blue stain) for 5 min at room temperature, and viability was assessed by counting viable and unviable cells in a Neubauer chamber.

### J774 A.1 mouse macrophage cell culture and infection with *Paracoccidioides*

The J774 A.1 mouse macrophage cells were purchased from a cell bank in Rio de Janeiro (Rio de Janeiro, Brazil) and were maintained in cell culture bottles in an atmosphere of 5% carbon dioxide at 37°C in RPMI 1640 medium supplemented with 10% heat-inactivated fetal bovine serum (Vitrocell/Embriolife, Campinas, SP, Brazil), 10% amino acid solution (Sigma Biochemical, St. Louis, MO, USA) and 0.2% of gentamicin solution (Sigma Biochemical).

The macrophages were quantified by counting them in a Neubauer chamber, after which they were plated at a density of 10^6^ cells per well on glass cover slips in 24-well culture plates. The macrophages were then infected with 2.5 × 10^6^*Paracoccidioides Pb*01 yeast cells, also determined by counting Neubauer chamber. The cells were co-cultured for 24 h in the presence of 0.32 M OenB at 36°C and 5% CO_2_ to allow the internalization of the fungus. After this period, the supernatants were then aspirated, and the layer was observed microscopically, to visualize the fungal cells that had been internalized by the macrophages. The number of viable fungi co-cultivated with the macrophages was determined by quantifying the number of colony forming units (CFUs). Then, the monolayer was gently washed with 1× PBS to remove any non-adherent/internalized yeast cells, and the total RNA was isolated.

### RNA isolation and cDNA synthesis

*Paracoccidioides Pb*01 yeast cells were cultured in MMcM medium only or in the same medium with 0.32 M OenB for 90 and 180 min. For RNA isolation, cells were collected by centrifugation and the RNAs of driver and tester cultures were extracted with Trizol (Invitrogen, Carlsbad, CA, USA) according to the manufacturer’s instructions. RNA quality was assessed by determining the A_260nm_/A_280nm_ ratio and by the visualization of RNAs by 1.5% agarose gel electrophoresis. The RNAs were used to construct subtracted cDNA libraries. The first-strand cDNAs were synthesized from 1 μg of total RNAs using reverse transcriptase (RT Superscript III, Invitrogen) and were then used as a template from which to synthesize the second-strand using a SMART PCR cDNA synthesis kit (Clontech Laboratories, Palo Alto, CA, USA).

## RDA method

The RDA technique was used to generate libraries [27]. RDA is a PCR-based subtractive enrichment procedure. Originally developed for the identification of differences between complex genomes [82], this technique was adapted to enable the isolation of genes with an altered expression for comparison among various cell samples (cDNA RDA) [83]. The procedure relies on the generation of cDNA fragments from two different mRNA populations digested with restriction endonucleases, followed by adapter ligation and PCR amplification. The generated fragments are then subjected to successive rounds of subtractive hybridization and selective PCR amplification, to enrich the fragments of cDNA that are more abundant in one population [27].

The double-stranded cDNAs (1 μg) were then digested with the *Sau3*AI restriction enzyme (Amersham Biosciences, Little Chalfont, Uppsala, Sweden), and the digestion products were linked to adapter primers and PCR-amplified to generate cDNAs representative of the driver and tester libraries. Two successive rounds of PCR amplification employing different adapters (J-Bam and N-Bam, Table 2) were performed to enrich the differentially expressed sequences. In the first round, the tester/driver ratio of 1:10 was used for the hybridization, whereas in the second round the tester/driver ratio was set at a higher stringency of 1:100. Before each round of hybridization, the cDNAs were purified using a GFX PCR purification kit (GE Healthcare UK, Little Chalfont, Buckinghamshire, England). In the RDA method, cDNA from yeast cells cultured in MMcM broth medium containing 0.32 M of OenB for 90 and 180 min was used as the tester population and was hybridized to cDNA from yeast cells cultured in MMcM broth medium only for 90 and 180 min as the driver population, and vice-versa. This resulted in four differential expression libraries, two for up-regulated and two for down-regulated transcripts. The adapters used for the subtractive hybridizations are listed in Table 2.

The cDNAs from these libraries were cloned into pGEM-T Easy vector (Promega, Madison, WI, USA), which was used to transform *Escherichia coli* XL1 Blue electro competent cells. The positive colonies were picked and grown in deep-well plates. The plasmid DNA was purified by a miniprep protocol and was used as the template in the standard fluorescence labeling dye-terminator protocols with a T7 flanking vector primer. The reaction products were loaded onto a MegaBACE 1000 DNA sequencing system (GE Healthcare) for automated sequence analysis.

### Bioinformatics analysis

The reading quality was checked by the Phred program module [84], and were chose to analyze the sequences with at least 50 nucleotides and a quality grater or equal to 20. Subsequently, vector sequences were trimmed using the Crossmatch program [85]. Readings that had passed the quality check were next submitted to a CAP3 program [86] to obtain the final set of contigs and singlet sequences. All these tools were integrated in a specific pipeline [87]. The sequences obtained were dynamically translated and compared against the GenBank [88] non-redundant (nr) database from NCBI using the Blast X program [89] and the nucleotide database from the *Paracoccidioides* structural genome [90]. The database sequence matches were considered significant at *e-values* ≤10^-5^.

Sequence analysis and the annotation pipeline were set up using the Blast2GO program [91] that joints in one GO application based on a similarity search with statistical analysis and highlighted visualization on a directed acyclic graph [92]. The Blast2GO annotation algorithm already took multiple parameters into account such as sequence similarity, blast HSP (highest scoring pair) length and *e-values*, the GO hierarchical structure and GO term evidence codes [92,93]. The sequences were grouped into functional categories according to the classification of the MIPS functional catalog [94].

Graphs were plotted to demonstrate the statistically enriched GO functions with up- or down-regulated genes, respectively, for the times of exposure to the compound (Additional file 1: Figure S1). The percentage of occurrence of each gene in relation to the total number of genes from the libraries was calculated and shown Additional file 2: Table S1.

### Quantitative real-time PCR

For the qRT-PCR analysis of a subset of genes indicated as differentially expressed, gene-specific primers were designed using the software Primer Express (Applied Biosystems, Foster City, CA, USA). The predicted product lengths varied between 100 bp and 200 bp. The reference α-tubulin gene was used for normalization. Total RNA was extracted from control and OenB-treated yeast using Trizol reagent. The total RNA was also extracted from control and OenB-treated cells after infection with *Paracoccidioides*.

First strand cDNA was produced using a SuperScript III (Invitrogen) and oligo (dT)_15_ primer. All qRT-PCR assays were run using a SYBR Green (Applied Biosystems) protocol in a StepOnePlus^TM^ Real-Time PCR system (Applied Biosystems). The amplification protocol was 40 cycles of 95°C for 15 s; 60°C for 1 min. The qRT-PCR assays were performed in triplicate.

The SYBR Green PCR master mix (Applied Biosystems) was used as the reaction mixture, and 10 pmol of each specific primer and 40 ng of template cDNA were added to a final volume of 25 μL. A melting curve analysis was performed to confirm a single PCR product. The data were normalized against α-tubulin in each set of qRT-PCR experiments. The relative expression levels of the genes of interest were calculated using the standard curve method for relative quantification [95]. The Student’s *t* test was used for statistical comparisons and *P values* ≤ 0.05 were considered statistically significant. The specific sense and antisense primers are listed in Table 2.

### Dosage of cell wall polymers

To measure the amount of total carbohydrates, *Paracoccidioides Pb*01 yeast cells were grown in liquid Fava-Netto’s medium at 36°C for 72 h with or without OenB. The cell wall polymers were obtained as previously described [29]. In brief, yeast cells were collected and washed with acetone, ethyl alcohol and ether. Then, the cells were suspended in 50 mM TrisHCl pH 7.5 and disrupted with glass beads. Cell walls were separated from the cytosolic fraction by centrifugation. The alkali-soluble (AS) fraction was extracted with NaOH and sodium borohydride. Total carbohydrates were determined by the phenol sulfuric acid procedure. The amount of 1,3-β-D-glucan in the AI fraction was estimated by measuring the release of reducing sugars.

The amount of N-acetylglucosamine was determined as previously described [29]. Briefly, the *Paracoccidioides Pb*01 yeast cells enriched cell wall fraction was subsequently hydrolyzed in HCl and neutralized with NaOH. The sample was added to solution A and followed by solution B. Absorbance at 520 nm was measured and compared to absorbance values from a standard curve of glucosamine taken through the same reactions.

### Fungus cell wall integrity analysis

CFW and CR (Sigma Biochemical) were utilized to stain the *Paracoccidioides Pb*01 and *Pb*18 cell wall*,* in order to show the effect of OenB by means of the fluorescence of the stain. *Paracoccidioides* was grown in liquid Fava-Netto’s medium at 36°C for 72 h with or without 0.32 M of OenB. Briefly, the cells were fixed in 100% methanol at -80°C for 20 min and then at -20°C for 20 min, and they were then washed and centrifuged. The cells were collected, stained with 100 μg/mL CFW and CR in PBS for 15 min and washed with PBS. The specimens were analyzed under a fluorescence microscope (Zeiss Axiocam MRc – Scope A1).

### RDA libraries data accession

The RDA data from this study is available on the EST database (dbEST) at http://www.ncbi.nlm.nih.gov/nucest/?term= under series LIBEST_028147 *Paracoccidioides* oenoteinB 90 up Library, LIBEST_028146, *Paracoccidioides* oenoteinB 90 down Library, LIBEST_028149 *Paracoccidioides* oenoteinB 180 up Library and LIBEST_028148 *Paracoccidioides* oenoteinB 180 down Library.

## Authors’ contributions

PFZC conducted all the experiments described in the manuscript; SCS and PHF provided extracts of oenothein B for experiments; PKT participated in the polymer dosage experiments; CLB contributed to the RDA experiments; WSM contributed to the bioinformatic analysis; CMAS and MP contributed to the preparation of the manuscript. MP designed the study, provided support and co-wrote the manuscript. All authors contributed to the discussion of results. All authors have read and approved the final manuscript.

## Competing interests

The authors declare that they have no competing interests.

## Supplementary Material

Additional file 1: Figure S1 Graph plotted to demonstrate the statistically enriched GO functions with up- or down-regulated genes after exposure to the Oen B. Functional classification of *Paracoccidioides* cDNAs derived from RDA experiments. The percentage of each functional category is shown. The percentage of occurrence of each gene in relation to the total number of genes from the libraries was calculated and shown Additional file 2: Table S1. The functional classification was based of MIPS functional annotation scheme. Each functional class is represented as a color-coded segment and expressed as a percentage of the total number of ESTs in each library. Click here for file

Additional file 2: Table S1 Percentage of ESTs up and down regulated genes of *Paracoccidioides* yeast cells treated with OenB by 90 and 180 min. Click here for file
