# Supplementary figures and images for: Transcriptional profile of Paracoccidioides induced by oenothein B, a potential antifungal agent from the Brazilian Cerrado plant Eugenia uniflora
Source: BMC Microbiol. 2013 Oct 12;13:227. doi: 10.1186/1471-2180-13-227 (PMC3852496; doi:10.1186/1471-2180-13-227)

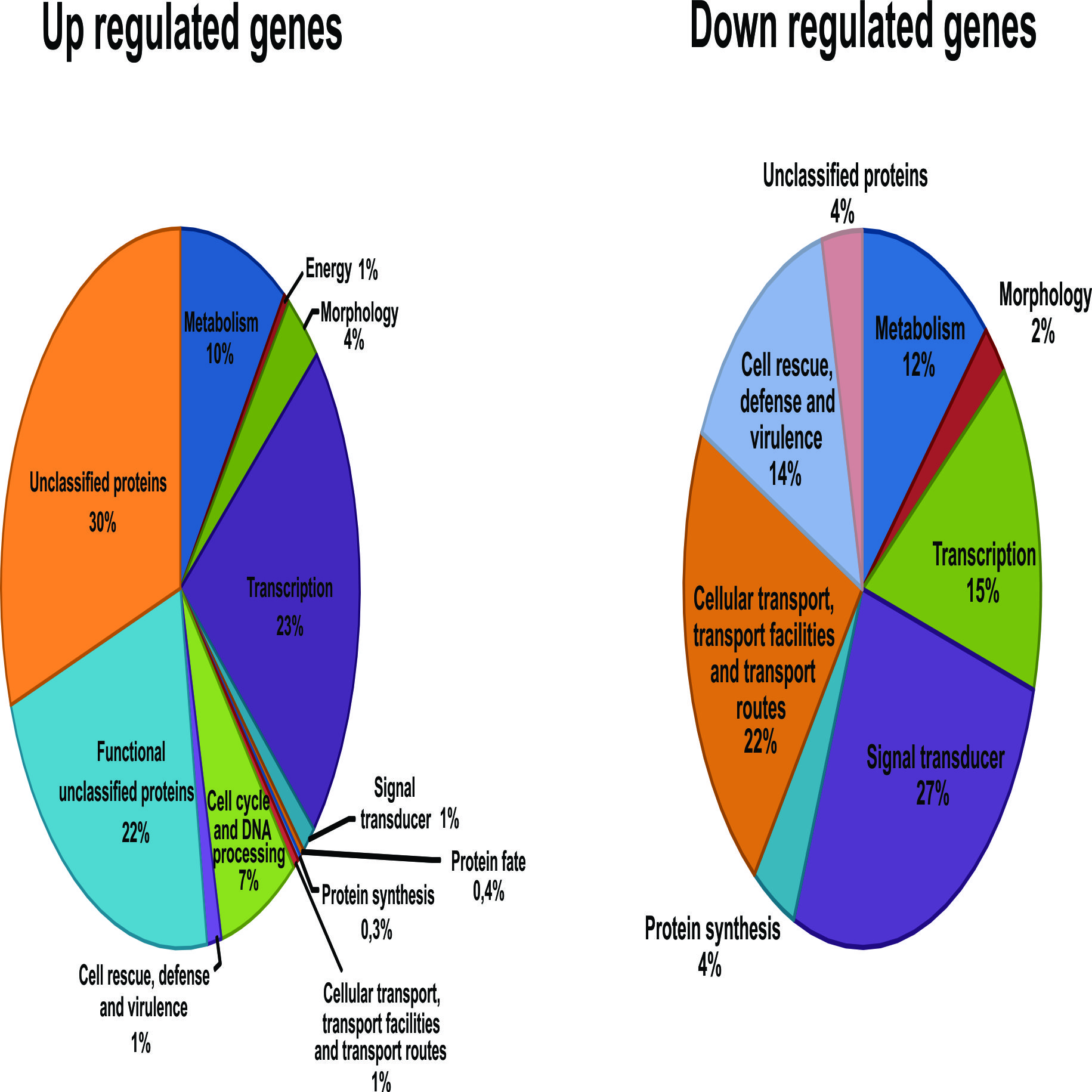

Supplement: Additional file 1: Figure S1 — Graph plotted to demonstrate the statistically enriched GO functions with up- or down-regulated genes after exposure to the Oen B. Functional classification of Paracoccidioides cDNAs derived from RDA experiments. The percentage of each functional category is shown. The percentage of occurrence of each gene in relation to the total number of genes from the libraries was calculated and shown Additional file 2: Table S1. The functional classification was based of MIPS functional annotation scheme. Each functional class is represented as a color-coded segment and expressed as a percentage of the total number of ESTs in each library. [file 1471-2180-13-227-S1.jpeg]
