# Supplementary material for: Transcriptional profile of Paracoccidioides induced by oenothein B, a potential antifungal agent from the Brazilian Cerrado plant Eugenia uniflora
Source: BMC Microbiol. 2013 Oct 12;13:227. doi: 10.1186/1471-2180-13-227 (PMC3852496; doi:10.1186/1471-2180-13-227)
Supplement: Additional file 2: Table S1 — Percentage of ESTs up and down regulated genes of Paracoccidioides yeast cells treated with OenB by 90 and 180 min. [file 1471-2180-13-227-S2.docx]

**Additional file 2: Table S1** Percentage of ESTs up and down regulated genes of *Paracoccidioides* yeast cells treated with OenB by 90 and 180 min.

| **Gene product** | ***Paracoccidioides***  **acession number^a^** | **Percentage of occurrence** | | | |
| --- | --- | --- | --- | --- | --- |
|  |  | **90 min** | | **180 min** | |
|  |  | **up** | **down** | **up** | **down** |
| Alanine glyoxylate aminotransferase **(AGX1)** | PAAG_03138.1 | 0.43 |  |  | 0.70 |
| Hexokinase 1 **(HXK1)** | PAAG_01377.1 | 0.43 |  |  |  |
| Urease **(URE)** | PAAG_00954.1 | 0.21 |  |  |  |
| Betaine aldehyde dehydrogenase **(BADH)** | PAAG_05392.1 |  | 1.92 |  | 11.20 |
| β glucosidase **(BGLU)** | PAAG­_04545.1 | 0.21 |  |  |  |
| Cysteine desulfurase **(NFS1 )** | PAAG_05850.1 | 0.86 |  |  | 4.90 |
| Trehalose phosphatase **(TPS1)** | PAAG_06703.1 | 4.32 |  |  |  |
| C-5 sterol desaturase **(ERG 3)** | PAAG_03651.1 |  | 3.85 |  |  |
| NADP-dependent leukotriene B4 12-hydroxydrogenase **(LTB_4_DH)** | PAAG_05416.1 | 0.21 |  |  |  |
| Fatty acid elongase **(GNS1/SUR4)** | PAAG_08553.1 | 0.43 |  |  |  |
| Glutamine synthetase **(GLN1)** | PAAG_07003.1 | 2.38 |  | 10.30 |  |
| Acyl-CoA dehydrogenase **(ACAD)** | PAAG_03490.1 | 0.21 |  |  |  |
| Glucose methanol choline oxidorreductase **(GMCO)** | PAAG_08146.1 |  |  | 0.99 |  |
| β glucan synthesis associated protein **(KRE6)** | PAAG_00091.1 |  |  | 0.99 |  |
| α 1,6 mannosyltransferase **(OCH1)** | PAAG_01658.1 | 1.08 |  |  |  |
| Arp 2/3 complex subunit Arc16 | PAAG_03624.1 |  |  | 3.99 |  |
| Cell morphology protein **(PAL1)** | PAAG_02031.1 | 0.21 |  |  |  |
| Integral membrane protein **(MPV17/PMP22)** | PAAG_02868.1 |  | 5.80 |  |  |
| GYF domain protein | PAAG_00627.1 | 1.08 |  |  |  |
| C_2_H_2_ transcription factor **(SEB1)** | PAAG_03287.1 | 0.43 |  |  |  |
| C6 transcription factor **(CTF1B)** | PAAG_01359.1 |  | 3.24 |  |  |
| Fork head box protein D1 **(FOXD1)** | PAAG_07388.1 | 0.21 |  |  |  |
| GATA type sexual **(NSDD)** | PAAG_05818.1 | 4.75 |  |  | 2.80 |
| Histone deacetylase **(RPD3)** | PAAG_06742.1 | 2.60 |  |  |  |
| RING finger protein **(RNF)** | PAAG_06129.1 |  | 2.88 |  | 10.50 |
| Transcription factor **(ATF1)** | PAAG_01945.1 | 0.21 |  |  |  |
| Transcription factor fungi | PAAG_02049.1 |  |  | 38.54 |  |
| Protein RNP domain | PAAG_03136.1 | 0.43 |  |  |  |
| Transcription factor prr1**(HSF1)** | PAAG_05064.1 | 0.21 |  |  |  |
| Transcription factor **(STEA)** | PAAG_00406.1 | 3.90 |  |  |  |
| Guanine nucleotide binding protein alpha-1 subunit **(GPA2)** | PAAG_04436.1 |  | 22.11 |  |  |
| Protein with PYP-likesensor domain **(PAS)** | PAAG_06301.1 | 2.20 |  |  | 30.07 |
| Proteasome component **(PRE6)** | PAAG_07802.1 |  |  | 0.99 |  |
| Elongation factor 1-gamma 1 **(eEF-1)** | PAAG_03556.1 |  |  |  | 0.70 |
| ATP-dependent RNA helicase **(eIF-4A)** | PAAG_00689.1 | 0.43 |  |  | 6.30 |
| Calcium-transporting ATPase sarcoplasmic/endoplamic reticulum type **(PMR1)** | PAAG_00774.1 | 0.21 |  |  |  |
| Ferric-chelate reductase **(FRE)** | PAAG_05370.1 |  |  | 0.66 |  |
| Succinate/fumarate mitochondrial transporter **(SFC1)** | PAAG_06563.1 |  | 3.85 |  |  |
| Membrane biogenesis protein **(YOP1)** | PAAG_00481.1 |  |  | 0.33 |  |
| Major facilitator superfamily transporter **(MFS)** | PAAG_01353.1 |  | 35.60 |  | 6.30 |
| Bodown 198 – Major facilitador superfamily transporter | PAAG_06077.1 |  | 3.85 |  |  |
| Ribonuclease reductase large subunit **(RNR1)** | PAAG_02210.1 | 0.21 |  |  |  |
| Arginine N-methyltransferase - SKB1 **(PRMT5)** | PAAG_02402.1 | 11.23 |  |  |  |
| SH3 domain-containing protein **(CYK3)** | PAAG_02301.1 | 0.43 |  |  |  |
| Phosphatase regulatory subunit **(GAC1)** | PAAG_00128.1 | 2.20 |  |  |  |
| Heat shock protein **(HSP70)** | PAAG_08003.1 |  | 0.96 |  |  |
| Pathogenesis associated protein **(CAP20)** | PAAG_06538.1 |  |  |  | 23.78 |
| Uncharacterized protein family UPF0121 | PAAG_00184.1 |  |  | 1.33 |  |
| Pleckstrin Homology (PH) domain | PAAG_03092.1 | 2.40 |  |  |  |
| DUF 1688 domain protein | PAAG_04190.1 | 33.26 |  |  |  |
| Conserved hypothetical protein | PAAG_06834.1 | 5.40 |  |  |  |
| Conserved hypothetical protein | PAAG_07365.1 | 7.34 |  |  |  |
| Conserved hypothetical protein | PAAG_05009.1 | 2.20 |  |  | 2.10 |
| Conserved hypothetical protein | PAAG_07364.1 | 0.86 |  | 0.66 |  |
| Conserved hypothetical protein | PAAG_04732.1 | 1.51 |  |  |  |
| Conserved hypothetical protein | PAAG_03559.1 | 0.43 |  |  |  |
| Conserved hypothetical protein | PAAG_02868.1 | 2.38 |  |  |  |
| Conserved hypothetical protein | PAAG_01170.1 | 0.21 |  |  |  |
| Conserved hypothetical protein | PAAG_04190.1 |  | 0.96 |  |  |
| Conserved hypothetical protein | PAAG_06925.1 |  |  | 0.33 |  |
| Conserved hypothetical protein | PAAG_07770.1 |  |  | 0.33 |  |
| Conserved hypothetical protein | PAAG_05850.1 |  | 0.96 |  |  |
| Conserved hypothetical protein | PAAG_06301.1 |  |  | 0.33 |  |
| Conserved hypothetical protein | PAAG_07127.1 |  | 2.88 |  |  |
| Hypothetical protein | PAAG_07199.1 |  |  | 22.92 |  |
| Hypothetical protein | PAAG_03580.1 | 1.30 |  |  |  |
| Hypothetical protein | PAAG_04733.1 | 0.65 |  |  |  |
| Hypothetical protein | PAAG_06820.1 | 0.21 |  |  |  |
| Hypothetical protein | PAAG_05558.1 |  |  | 0.33 |  |
| Hypothetical protein | PAAG_03580.1 |  |  |  | 0.33 |
| Hypothetical protein | PAAG_03099.1 |  |  | 14.95 |  |
| Predicted protein | PAAG_01169.1 | 0.21 |  |  |  |
| Predicted protein | PAAG_05526.1 |  |  | 1.66 |  |
| Predicted protein | PAAG_03257.1 |  |  | 0.33 |  |

^a^ Accesion number at Broad (http://www.broadinstitute.org).
